# Incidence and characteristics of eravacycline-associated increase in serum bilirubin levels: a retrospective study

**DOI:** 10.3389/fphar.2026.1585390

**Published:** 2026-01-19

**Authors:** Yueyue Si, Man Chen, Jicheng Zhang, Jinjiao Jiang, Yahui Zhang, Guofeng Yu, Nan Guo, Genquan Yan, Bing Leng

**Affiliations:** 1 Department of Pharmacy, Shandong Provincial Hospital Affiliated to Shandong First Medical University, Jinan, China; 2 Department of Pharmacy, Yantai City Yantaishan Hospital, Yantai, China; 3 Department of Critical Care Medicine, Shandong Provincial Hospital Affiliated to Shandong First Medical University, Jinan, China; 4 Yantai Service Center for Drug Evaluation and Inspection, Yantai, China

**Keywords:** eravacycline, tigecycline, total bilirubin, adverse reaction, retrospective study

## Abstract

**Objectives:**

To compare the effects of eravacycline and tigecycline on serum bilirubin levels and investigate the incidence and clinical characteristics of total bilirubin increase associated with eravacycline.

**Methods:**

We conducted a retrospective study in critically ill patients receiving eravacycline or tigecycline between November 2023 and May 2024 to evaluate serum bilirubin levels. Causality was assessed using the Naranjo Adverse Drug Reaction Probability Scale. A multivariable logistic regression was conducted to identify the risk factors for an increase in total bilirubin, and Kaplan-Meier curves were used to depict the time to an increase in total bilirubin.

**Results:**

A total of 48 patients were evaluated in this study. Compared to tigecycline, eravacycline was associated with elevated serum total bilirubin and direct bilirubin. Following the definition of an increase in total bilirubin levels as ≥42 μmol/L, 15 patients were rated as possibly or probably having drug-associated increase in total bilirubin. The incidence of an increase in total bilirubin was 61.5% in the eravacycline group and only 20.0% in the tigecycline group. Administration of eravacycline was identified as an independent risk factor for an increase in total bilirubin. In addition, patients receiving eravacycline tended to experience an increase in total bilirubin significantly earlier, and the change in direct bilirubin levels was significantly greater than that in indirect bilirubin in these patients.

**Conclusion:**

Eravacycline has been identified as an independent risk factor for the increase in total bilirubin. Monitoring serum bilirubin levels should be considered in patients receiving eravacycline, particularly in critically ill patients.

## 1 Introduction

Infections caused by multidrug-resistant bacteria present a global challenge due to their rapid spread and the difficulty in treatment. As the issue continues to escalate, we may face a drug-free situation in the future. Consequently, the exploration and application of novel antimicrobial drugs have become necessary (Escola-Verge et al., 2020; van Duin and Paterson, 2020). Eravacycline is a novel class of fully synthetic tetracycline derivatives, with modifications to the D-ring of tigecycline (Sutcliffe et al., 2013). It was approved by the FDA for complicated intra-abdominal infections in adults in 2018, with a recommended dosage of 1 mg per kilogram administered intravenously every 12 h for a total of 4–14 days (Drugs and Lactation Database, 2020). Eravacycline exhibits broad-spectrum antimicrobial activity against Gram-positive, Gram-negative, and anaerobic bacteria by inhibiting bacterial protein synthesis through binding to the 30S ribosomal subunit. Several *in vitro* studies have reported that eravacycline has demonstrated activity against multidrug-resistant Gram-positive bacteria (methicillin-resistant *Staphylococcus aureus* and vancomycin-resistant *Enterococcus*) and multidrug-resistant Gram-negative bacteria (*Enterobacterales* that produce an extended-spectrum beta-lactamase, carbapenemase-resistant *Enterobacterales* and *Acinetobacter* species.) (Abdallah et al., 2015; Seifert et al., 2018; Morrissey et al., 2020; Ding et al., 2022; Hawser et al., 2023; Huang et al., 2023; Liao et al., 2024; Ataman and Celik, 2025). It has gained considerable attention as a therapeutic option in the context of the epidemic of multidrug-resistant Gram-negative bacilli. A retrospective study reported that eravacycline was commonly used as definitive therapy for infections caused by carbapenem-resistant *Acinetobacter baumannii and Enterobacterales* (Kunz Coyne et al., 2024).

With the rising use of eravacycline, concerns regarding its adverse effects have gradually emerged. The commonly observed adverse effects of eravacycline include gastrointestinal disturbances, nausea, and vomiting. A study utilizing data from FDA adverse event system suggested an association between drug-induced liver injury (DILI) and other tetracyclines, such as tigecycline, minocycline, and doxycycline (Wei et al., 2022). However, no such correlation has been identified for eravacycline, which is inconsistent with our clinical observation. To explore the impact of eravacycline on serum bilirubin levels, we conducted a retrospective study to compare the hepatic parameters in patients treated with eravacycline and tigecycline.

## 2 Materials and methods

### 2.1 Ethics

This study was conducted in accordance with the Declaration of Helsinki (2013) (World Medical Association, 2013). Ethics approval was obtained from the Medical Ethics Committee of Shandong Provincial Hospital affiliated to Shandong First Medical University (approval number SWYX2024-391). Informed consent was waived for the patients due to the retrospective design of the study. All personal information of the patients were anonymized during data collection and deidentified during data analysis.

### 2.2 Study design and patient selection

All patients who received either intravenous eravacycline (Xerava, Everest Medicines) or tigecycline (Tygacil, Pfizer) between November 2023 and May 2024 in the intensive care unit (ICU) of Shandong Provincial Hospital Affiliated to Shandong First Medical University were eligible for inclusion in the cohort. In patients who received multiple courses of eravacycline or tigecycline, only the first course was analyzed. Patients were excluded if they received eravacycline or tigecycline for a period <3 days, had a baseline total bilirubin (TBIL) ≥42 μmol/L (3 days before treatment), or lacked hepatic function data before and during therapy.

### 2.3 Retrospective study

A retrospective study design was employed. The study was conducted in two phases: Firstly, a cohort study was performed to compare basic information and laboratory parameters between patients treated with eravacycline and tigecycline. Subsequently, all patients were divided into two groups (an increase in TBIL group and a normal TBIL group) based on the level of bilirubin during treatment, and a case-control study was conducted to analyze data from them. The relationship between an increase in TBIL and patient demographics, comorbidities, important invasive procedures and medication was evaluated, based on clinical knowledge of potential risk factors.

### 2.4 Data collection

This retrospective analysis encompasses data on patient demographics (age and sex), APACHE II score, comorbidities (chronic liver disease, chronic kidney disease, diabetes mellitus, hypertension, atrial fibrillation, chronic heart failure, chronic obstructive pulmonary disease and malignancy), continuous renal replacement therapy [CRRT]), septic shock, duration, laboratory data (alanine aminotransferase [AST], aspartate aminotransferase [ALT], total bilirubin [TBIL], direct bilirubin [DBIL], indirect bilirubin [IDIL], albumin [ALB], creatinine [Cr]). All data above were obtained from the patient’s medical records.

An increase in TBIL was defined as a value ≥ 2 times the upper limit of normal (42 μmol/L) (normal TBIL range 0–21 μmol/L), referring to the Chinese guideline for diagnosis and management of drug-induced liver injury (2023 version) (Mao, 2023). The adverse drug reaction probability scale (Naranjo score) was conducted to evaluate the probability that an increase in TBIL was correlated with tigecycline or eravacycline use (Naranjo et al., 1981).

### 2.5 Statistical analysis

Continuous variables with a normal distribution were analyzed using the t-test, and the results are presented as mean ± standard deviation. For continuous variables with non-normally distributed data, non-parametric tests were applied, with results shown as median (interquartile range [IQR]). Categorical variables were expressed as ratios and analyzed using the χ2 or Fisher’s exact test. The time to an increase in TBIL between the two cohorts was compared using the Kaplan–Meier survival analysis and log-rank test. Variables that showed statistical significance in univariate analyses were entered into a multivariate logistic regression model to identify risk factors for an increase in TBIL, with odds ratios (ORs) and 95% confidence intervals (CIs) calculated. A two-tailed p-value of *P* < 0.05 was considered statistically.

## 3 Results

### 3.1 Demographics and clinical characteristics

**FIGURE 1:**
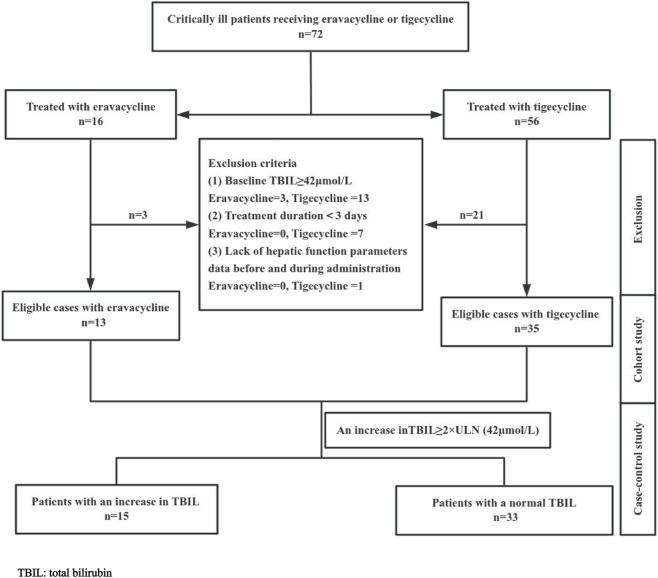
Flow diagram of the study selection process. There were 72 patients receiving eravacycline or tigecycline, while only 48 patients met the criteria for inclusion in this study.

A total of 72 patients were treated with eravacycline or tigecycline in the ICU from November 2023 to May 2024. Of these, 48 patients were eligible for inclusion in the study, comprising 13 patients receiving eravacycline and 35 patients receiving tigecycline (Figure 1). As illustrated in Table 1, the mean age of the 48 patients was 51.29 ± 17.11 years, with 20.8% being female. The mean duration of antibiotic administration was 9.42 ± 4.76 days. There were no significant differences in age, gender, APACHE II score, comorbidities (chronic liver disease, chronic kidney disease, diabetes mellitus, hypertension, atrial fibrillation, chronic heart failure, chronic obstructive pulmonary disease and malignancy), CRRT treatment, septic shock, or duration of medication between the two cohorts.

**TABLE 1:** Patient characteristics.

Variable^a^	Total (n = 48)	Eravacycline (n = 13)	Tigecycline (n = 35)	*P* value^b^
Age (years)	51.29 ± 17.11	46.77 ± 16.55	52.97 ± 17.25	0.269
Sex, female (%)	10 (20.8%)	2 (15.4%)	8 (22.9%)	0.868
APACHE II score	18.00 (12.50–20.00)	17.00 (13.50–20.00)	18.00 (12.00–20.00)	0.852
Comorbidities (%)				
Chronic liver disease	3 (6.3%)	1 (7.7%)	2 (5.7%)	1.000
Chronic kidney disease	3 (6.3%)	0 (0%)	3 (8.6%)	0.553
Diabetes mellitus	12 (25.0%)	2 (15.4%)	10 (28.6%)	0.574
Hypertension	18 (37.5%)	6 (46.2%)	12 (34.3%)	0.675
Atrial fibrillation	2 (4.2%)	0 (0%)	2 (5.7%)	1.000
Chronic heart failure	9 (18.8%)	3 (23.1%)	6 (17.1%)	0.959
Chronic obstructive pulmonary disease	1 (2.1%)	1 (7.7%)	0 (0%)	0.271
Solid malignancy	3 (6.3%)	1 (7.7%)	2 (5.7%)	1.000
Hematologic malignancy	1 (2.1%)	0 (0%)	1 (2.9%)	1.000
CRRT (%)	25 (52.1%)	8 (61.5%)	17 (48.6%)	0.424
Septic shock	25 (52.1%)	7 (53.8%)	18 (51.4%)	1.000
Duration (days)	9.42 ± 4.76	10.54 ± 5.14	9.00 ± 4.62	0.325

CRRT, continuous renal replacement therapy.

^a^ Mean (SD) or number (%), as appropriate.

^b^ Comparison between eravacycline and tigecycline: *t*-test for continuous variables, χ2 or Fisher’s exact test for categorical variables.

### 3.2 Comparision of the effects of eravacycline and tigecycline on hepatic parameters

The laboratory parameters of the two cohorts are presented in Table 2. There were no significant differences between the eravacycline and tigecycline groups in terms of baseline levels of hepatic and renal parameters (TBIL, DBIL, IBIL, AST, ALT, ALB and Cr).

**TABLE 2:** Comparision of laboratory parameters between eravacycline and tigecycline.

Variable^a^	Total (n = 48)	Eravacycline (n = 13)	Tigecycline (n = 35)	*P* value^b^
Baseline				
TBIL (μmol/L)	15.96 (12.22–26.66)	19.85 (15.08–28.52)	15.10 (11.02–25.34)	0.135
DBIL (μmol/L)	6.34 (3.05–11.62)	9.10 (5.42–12.75)	5.00 (2.90–10.24)	0.123
IBIL (μmol/L)	10.47 (8.05–14.95)	11.39 (8.57–16.59)	9.56 (7.82–14.60)	0.286
AST (U/L)	40.00 (27.00–59.00)	32.00 (21.50–63.00)	42.00 (27.75–59.25)	0.379
ALT (U/L)	28.00 (15.50–58.00)	26.00 (13.50–69.00)	36.00 (17.00–58.00)	0.610
ALB (g/L)	33.35 (30.85–36.88)	33.20 (29.95–35.05)	34.10 (30.80–37.50)	0.546
Cr (μmol/L)	91.85 (62.93–161.89)	96.00 (67.15–194.55)	80.57 (54.03–135.09)	0.359
After administration				
TBIL (μmol/L)	28.21 (17.22–53.33)	53.62 (22.82–116.75)	22.70 (16.58–37.63)	0.022
DBIL (μmol/L)	11.21 (5.36–26.90)	32.03 (7.45–70.65)	9.80 (4.78–18.40)	0.009
IBIL (μmol/L)	16.35 (11.54–28.40)	21.91 (12.61–46.10)	13.46 (11.32–20.27)	0.072
AST (U/L)	37.00 (27.25–63.75)	39.00 (29.50–135.00)	36.00 (27.00–56.00)	0.291
ALT (U/L)	27.50 (17.00–60.50)	35.00 (18.50–98.50)	27.00 (15.00–47.00)	0.403
ALB (g/L)	34.10 (30.10–37.35)	32.40 (27.40–41.50)	34.50 (31.00–37.20)	0.539

AST, alanine aminotransferase; ALT, aspartate aminotransferase; TBIL, total bilirubin; DBIL, direct bilirubin; IDIL, indirect bilirubin; ALB, albumin; Cr, creatinine.

^a^ Median (IQR).

^b^ Comparison between eravacycline and tigecycline: Mann–Whitney for continuous variables.

After administration, the serum TBIL and DBIL in the eravacycline group were significantly higher than those in the tigecycline group (53.62 μmol/L vs. 22.70 μmol/L, *P* = 0.022; 32.03 μmol/L vs. 9.80 μmol/L, *P* = 0.009, respectively). However, serum IBIL between the two groups did not show significant difference, although the median in the eravacycline group was higher (21.91 μmol/L vs. 13.46 μmol/L, *P* = 0.072). There were also no significant differences in other hepatic parameters (AST, ALT, ALB).

According to the definition of an increase in TBIL, Naranjo criteria indicated that 15 patients were possibly or probably associated with an increase in TBIL, 8 receiving eravacycline and 7 receiving tigecycline respectively. The incidence of an increase in TBIL was significantly higher in the eravacycline group compared to the tigecycline group (8/13 [61.5%] vs. 7/35 [20.0%], *P* = 0.012). The relative risk (RR) of developing an increase in TBIL with eravacycline versus tigecycline was 3.077 (95% CI: [1.397 to 6.778]).

### 3.3 Risk factors for an increase in TBIL

A case-control study was conducted to compare 15 patients with an increase in TBIL and 33 patients with normal TBIL levels. No significant differences in the demographic and clinical characteristics were found between the two groups, except for septic shock (80.0% vs. 39.4%, *P* = 0.009), CRRT (80.0% vs. 39.4%, *P* = 0.009), and administration of eravacycline (53.3% vs. 15.2%, *P* = 0.012). These variables with significance of *P* < 0.05 in univariate analyses were incorporated into multivariate analysis. Finally, only selection of eravacycline (OR 9.827, 95% CI:1.641–58.841, *P* = 0.012) was identified as independent risk factor for an increase in TBIL, by multiple logistic regression analysis (Table 3).

**TABLE 3:** Comparison of demographic and clinical characteristics of patients with high and normal TBIL during administration.

Variable^a^	Total (n = 48)	Increase in TBIL (n = 15)	Normal TBIL (n = 33)	Univariate analysis	Multivariate logistic regression analysis	
				*P* value	OR (95%CI)	*P* value^b^
Age (years)	51.29 ± 17.11	48.93 ± 14.28	52.36 ± 18.36	0.526	​	​
Sex, female (%)	10 (20.8%)	3 (20.0%)	7 (21.2%)	1.000	​	​
APACHE II score	18.00 (12.50–20.00)	20.00 (15.00–25.00)	18.00 (12.00–19.50)	0.074	​	​
Comorbidities (%)						
Chronic liver disease	3 (6.3%)	2 (13.3%)	1 (3.0%)	0.227	​	​
Chronic kidney disease	3 (6.3%)	1 (6.7%)	2 (6.1%)	1.000	​	​
Diabetes mellitus	12 (25.0%)	2 (13.3%)	10 (30.3%)	0.292	​	​
Hypertension	18 (37.5%)	7 (46.7%)	11 (33.3%)	0.376	​	​
Atrial fibrillation	2 (4.2%)	1 (6.7%)	1 (3.0%)	0.532	​	​
Chronic heart failure	9 (18.8%)	3 (20%)	6 (18.2%)	1.000	​	​
Chronic obstructive pulmonary disease	1 (2.1%)	0 (0.0%)	1 (3.0%)	1.000	​	​
Solid malignancy	3 (6.3%)	1 (6.7%)	2 (6.1%)	1.000	​	​
Hematologic malignancy	1 (2.1%)	0 (0.0%)	1 (3.0%)	1.000	​	​
CRRT (%)	25 (52.1%)	12 (80.0%)	13 (39.4%)	0.009	3.749 (0.681–20.640)	0.129
Septic shock	25 (52.1%)	12 (80.0%)	13 (39.4%)	0.009	5.977 (0.948–37.666)	0.057
Duration (days)	9.42 ± 4.75	9.73 ± 5.47	9.27 ± 4.48	0.760	​	​
Administration of eravacycline (%)	13 (27.1%)	8 (53.3%)	5 (15.2%)	0.012	9.827 (1.641–58.841)	0.012

CRRT, continuous renal replacement therapy.

^a^ Mean (SD) or number (%), as appropriate.

^b^ Comparison between eravacycline and tigecycline: *t*-test for continuous variables, χ2 or Fisher’s exact test for categorical variables.

### 3.4 Time and severity of onset of an increase in TBIL

**FIGURE 2:**
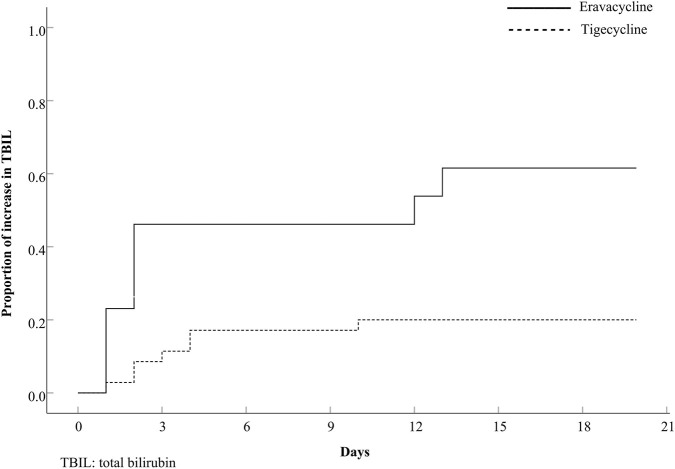
Comparison of onset of increase in total bilirubin (TBIL). *P* = 0.003. The broken line represents patients with tigecycline and the continuous line represents patients with eravacycline.

For the 15 patients with an increase in TBIL, the time to an increase in TBIL was significantly earlier in the eravacycline-exposed patients (*P* = 0.003), as shown in Figure 2, which depicts Kaplan–Meier curves for an increase in TBIL in eravacycline group versus tigecycline group. The incidence of an increase in TBIL during the first 3 days of exposure was significantly higher in the eravacycline group compared to the tigecycline group (46.2% vs. 11.4%, *P* = 0.016).

**FIGURE 3:**
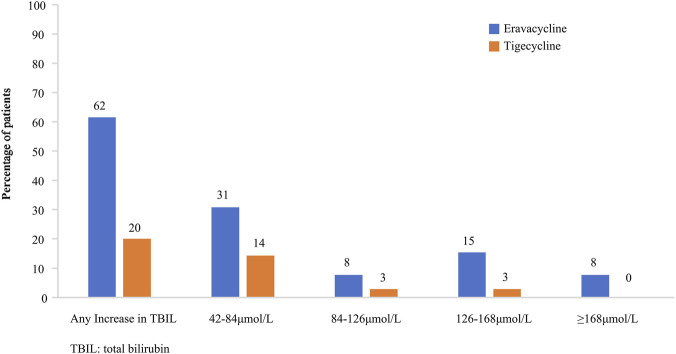
Severity of increase in total bilirubin (TBIL) of eravacycline and tigecycline.

The severity of an increase in TBIL in the two cohorts is depicted in Figure 3. 31% of eravacycline-exposed patients and 14% of tigecycline-exposed patients had TBIL levels within the range of 42–84 μmol/L (2–4×ULN); while within the range of ≥126 μmol/L (6×ULN), the proportions were 23% and 3%, respectively. The highest value of TBIL was 352.63 μmol/L in eravacycline group and 148.53 μmol/L in tigecycline group.

### 3.5 Changes in serum DBIL and IBIL levels

**FIGURE 4:**
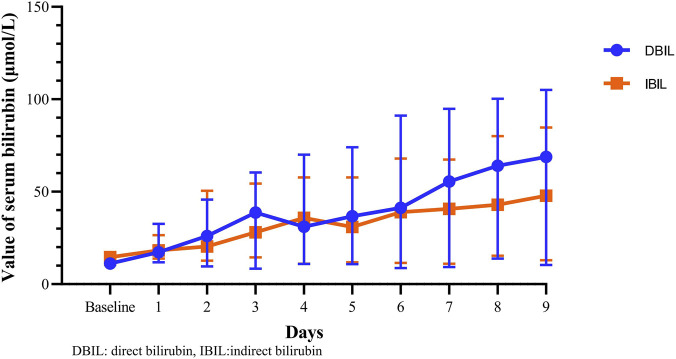
The changing trends in serum direct bilirubin (DBIL) and indirect bilirubin (IBIL) levels. Blue dots and orange rectangles represent the median value, whiskers represent the interquartile range.

Of the eight eravacycline-exposed patients who presented with an increase in TBIL, DBIL value was significantly higher than IBIL (49.81 [33.02–85.04] vs. 42.45 [20.53–59.51], *P* = 0.017), in cases of similar baseline levels for both (11.12 [9.11–14.42] vs. 14.61 [10.51–17.73], *P* = 0.161). The change in DBIL levels from pre-treatment to treatment was significantly greater than one in IBIL (41.86 [24.34–71.86] vs. 27.85 [8.32–37.67], *P* = 0.017). The changing trends in serum DBIL and IBIL levels is shown in Figure 4.

## 4 Discussion

With a rising prevalence of multidrug-resistance bacteria, the new antibiotic eravacycline has emerged as a promising alternative attributed to its ability to evade common resistance mechanisms (Thaden et al., 2017). As a chemical derivative of tigecycline, eravacycline is considered to be more effective and safer than tigecycline (Zhanel et al., 2016; Guo et al., 2025). A previous study reported the occurrence of elevated bilirubin levels in healthy subjects receiving tigecycline (Yamashita et al., 2014). However, a more notable increase in bilirubin level was observed in critically ill patients exposed to eravacycline in this study. In detail, we found that serum TBIL and DBIL levels following eravacycline treatment were significantly higher in comparison to tigecycline treatment, which hinted the potential effect of eravacycline in increasing bilirubin levels. According to the definition of an increase in TBIL, the incidence of an increase in TBIL in patients receiving eravacycline was >60%, representing an approximately threefold higher risk compared to those treated with tigecycline. Furthermore, the multivariate logistic analysis proved that administration of eravacycline was a significant independent risk factor for an increase in TBIL in ICU patients.

Elevated serum bilirubin is an important marker of DILI, and tigecycline-associated DILI has been reported previously, with incidence of 5.7% in a 2021 study and 10.3% in a 2022 study (Shi et al., 2021; Yu et al., 2022). The greater impact of drug on serum bilirubin seemed to imply a greater potential to induce the hepatobiliary system injury (Korenblat and Berk, 2005). In addition, elevated bilirubin was also associated with a poor prognosis and increased risk of death in patients with severe sepsis and septic shock (Patel et al., 2015). Therefore, we recommend necessary attention be paid to serum bilirubin levels in patients receiving eravacycline, especially in those at potential risk of hepatobiliary system injury in ICU.

A further investigation was conducted to explore the time and severity of this adverse reaction in our study. Patients treated with eravacycline experienced a significantly earlier increase in TBIL than those treated with tigecycline, and the proportion of this increase reached at 46.2% within the first 3 days. Moreover, eravacycline had a more dramatic impact on the severity of an increase in TBIL than tigecycline did, with the former’s percentage being twice approximately that of the latter within the range of 2–4×ULN, and rising to 5-fold within the range of ≥6×ULN. In view of the above, the administration of eravacycline should be in accordance with the package insert or relevant guidelines. An assessment of hepatic function should be conducted prior to the administration of eravacycline, and it should be used with caution in patients with abnormal bilirubin levels. Even if the baseline bilirubin levels are within the normal range, it is still necessary to maintain continuous observation throughout the course of administration, including the early stage. Once abnormal bilirubin levels are detected, dosage adjustment, hepatoprotective agents, or withdrawal could be considered by physician.

Bilirubin comes from the breakdown of senescent red blood cells. IBIL (also unconjugated bilirubin) is conjugated with glucuronic acid in the hepatocyte to result in DBIL (also conjugated bilirubin), which is excreted in bile (Kwo et al., 2017). Analyzing the changes in different types of serum bilirubin, we found the change in DBIL was significantly greater than those in IBIL. It seems reasonable to conclude that the increase in TBIL is primarily due to an increase in DBIL, as TBIL is the sum of the DBIL and IBIL.

An elevated DBIL implies hepatocellular injury or cholestasis in most settings (Kwo et al., 2017). Previous studies have shown that increase in DBIL induced by lenalidomide is associated with intrahepatic cholestasis with bile duct dilatation or sinusoidal obstruction syndrome (Jena et al., 2012; Gildea and Roswarski, 2023). Antimicrobial drugs are a common cause of DILI, which can be classified as hepatocellular, cholestatic, or mixed, and cholestatic has been reported as the most common type of tigecycline-induced DILI (Maraqa et al., 2002; Russmann et al., 2005; Aygun et al., 2007; Shi et al., 2021; Yu et al., 2022). Histopathology revealed that tigecycline could cause cholestasis, micro cavitation, and punctate necrosis of liver cells, and tetracycline could cause bile duct paucity, severe cholestasis, and minimal necrosis and inflammation (Hunt and Washington, 1994; Shi et al., 2022). Like other tetracycline antibiotics, eravacycline is excreted via the biliary tract and tends to accumulate in bile, providing a favourable environment for cellular injury, which may partially explain the effect of eravacycline on serum bilirubin (Petraitis et al., 2018; Newman et al., 2019). The enhancement of effect of eravacycline on serum bilirubin might be related to the two alterations in the molecular structure of eravacycline (the pyrrolidine substituent at the C9 and the fluoro substituent at C7) compared to tigecycline. However, the precise mechanism by which eravacycline elevates bilirubin levels remains unclear, and further laboratory studies are required.

It should be noted that the present study has some limitations. Firstly, the relatively limited sample size with single-center design may have constrained the generalization of the conclusions. Secondly, the present study exclusively included critically ill patients, and the findings may not be directly extrapolated to non-critically ill populations. Additionally, we focused specifically on isolated bilirubin elevation associated with eravacycline, and therefore did not collect other liver function parameters such as alkaline phosphatase or perform systematic DILI evaluation. In future studies, we will incorporate comprehensive liver function testing to more thoroughly assess eravacycline’s hepatotoxic potential.

## 5 Conclusion

Our study identified the administration of eravacycline as an independent risk factor for the increase of TBIL. The incidence of an increase in TBIL in patients receiving eravacycline was >60%, with an approximately threefold higher risk compared to those receiving tigecycline. Therefore, monitoring serum bilirubin levels should be recommended for patients receiving eravacycline, particularly patients in ICU. However, further validation of these findings requires additional prospective studies.

## Data Availability

The original contributions presented in the study are included in the article/supplementary material, further inquiries can be directed to the corresponding author.
